# Statistical significance of cluster membership for unsupervised evaluation of cell identities

**DOI:** 10.1093/bioinformatics/btaa087

**Published:** 2020-03-06

**Authors:** Neo Christopher Chung

**Affiliations:** b1 Institute of Informatics, Faculty of Mathematics, Informatics, and Mechanics, University of Warsaw, Warsaw 02-097, Poland; b2 NHLBI Integrated Cardiovascular Data Science Training Program, University of California, Los Angeles, CA 90095, USA; b3 Departments of Physiology and Medicine (Cardiology), UCLA School of Medicine, Los Angeles, CA 90095, USA

## Abstract

**Motivation:**

Single-cell RNA-sequencing (scRNA-seq) allows us to dissect transcriptional heterogeneity arising from cellular types, spatio-temporal contexts and environmental stimuli. Transcriptional heterogeneity may reflect phenotypes and molecular signatures that are often unmeasured or unknown a priori. Cell identities of samples derived from heterogeneous subpopulations are then determined by clustering of scRNA-seq data. These cell identities are used in downstream analyses. How can we examine if cell identities are accurately inferred? Unlike external measurements or labels for single cells, using clustering-based cell identities result in spurious signals and false discoveries.

**Results:**

We introduce non-parametric methods to evaluate cell identities by testing cluster memberships in an unsupervised manner. Diverse simulation studies demonstrate accuracy of the *jackstraw* test for cluster membership. We propose a posterior probability that a cell should be included in that clustering-based subpopulation. Posterior inclusion probabilities (PIPs) for cluster memberships can be used to select and visualize samples relevant to subpopulations. The proposed methods are applied on three scRNA-seq datasets. First, a mixture of Jurkat and 293T cell lines provides two distinct cellular populations. Second, Cell Hashing yields cell identities corresponding to eight donors which are independently analyzed by the jackstraw. Third, peripheral blood mononuclear cells are used to explore heterogeneous immune populations. The proposed *P*-values and PIPs lead to probabilistic feature selection of single cells that can be visualized using principal component analysis (PCA), t-distributed stochastic neighbor embedding (t-SNE) and others. By learning uncertainty in clustering high-dimensional data, the proposed methods enable unsupervised evaluation of cluster membership.

**Availability and implementation:**

https://cran.r-project.org/package=jackstraw.

**Supplementary information:**

Supplementary data are available at *Bioinformatics* online.

## 1 Introduction

Single-cell RNA-seq (scRNA-seq) has enabled large-scale gene expression studies that help elucidate transcriptional heterogeneity related to cellular types, spatio-temporal contexts and environmental stimuli (Jaitin *et al.*, 2014; Macosko *et al.*, 2015; Patel *et al.*, 2014). Transcriptional heterogeneity is manifested on systematic variation across gene expression, which is characterized by unsupervised clustering. Clustering-based cell identities are used in downstream feature selection, differential expression analysis and visualization (Butler *et al.*, 2018; Guo *et al.*, 2015; Qiu *et al.*, 2017; Satija *et al.*, 2015). Given that cell identities are determined in an unsupervised manner, it is critical to evaluate if they are correctly assigned. We have developed novel methods to estimate statistical significances and posterior inclusion probabilities (PIPs) of assigning cell identities to estimated subpopulations. By learning uncertainty in applying clustering to scRNA-seq data, the proposed methods enable unsupervised evaluation of cluster memberships, such as cell identities.

Clustering has been one of the most popular analysis methods for high-dimensional genomic data. Gene expression studies have long used clustering to identify co-regulated subsets of genes (Eisen *et al.*, 1998; Gasch *et al.*, 2000; Spellman *et al.*, 1998) and subpopulations among samples (Alon *et al.*, 1999; Golub *et al.*, 1999; Sørlie *et al.*, 2001). Recently, there have been several scRNA-seq studies where gene expression from hundreds and thousands of single cells are measured en masse (Jaitin *et al.*, 2014; Macosko *et al.*, 2015; Zheng *et al.*, 2017). Identities of single cells are typically unknown a priori and characterized by unsupervised clustering. Clustering *m* cells to *K* subpopulations provides computationally defined *m* cell identities. These clustering-based cell identities are of great interests, as complex phenotypes and diseases may exhibit molecular signature as yet unknown.

Single-cell analysis tools implement various clustering algorithms, including, but not limited to, K nearest neighbors in Seurat (Butler *et al.*, 2018; Satija *et al.*, 2015), hierarchical clustering in SINCERA (Guo *et al.*, 2015) and density peak clustering in Monocle (Qiu *et al.*, 2017). Furthermore, a number of clustering algorithms specifically tailored to scRNA-seq data have been developed to identify subtypes of single cells (Buettner *et al.*, 2015; Wang *et al.*, 2017; Xu and Su, 2015; Zeisel *et al.*, 2015). To increase computational efficiency, a number of scRNA-seq studies combine principal component analysis (PCA) or t-distributed stochastic neighbor embedding (t-SNE; van der Maaten and Hinton, 2008) with unsupervised clustering (Macosko *et al.*, 2015; Satija *et al.*, 2015; Zheng *et al.*, 2017). Consensus (ensemble) algorithms combine multiple clustering results (Kiselev *et al.*, 2017; Yang *et al.*, 2018). What is overlooked in these recent developments is how to evaluate single cells, when their cell identities are determined by clustering. To the best of our knowledge, this represents the first study on estimating statistical significance of cluster membership at a single-cell level.

**Fig. 1.**
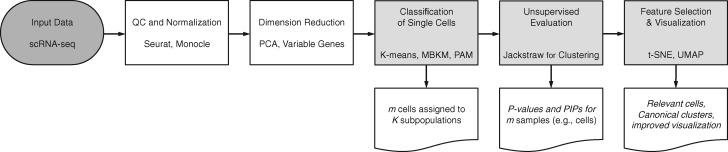
Analysis pipeline for scRNA-seq data for elucidating transcriptional heterogeneity. Without knowing cell identities, one may obtain gene expression profiles of single cells. After quality control, dimension reduction and unsupervised clustering are routinely applied to estimate cellular subpopulations that are used as cell identities in downstream analyses. The proposed methods enable statistically rigorous evaluation of cell identities improving unsupervised classification and feature selection

The proposed non-parametric methods leverage the assumption regarding cluster structure across single cells. Clustering algorithms estimate systematic variation and identify subsets of cells that contribute to distinct patterns. Due to high dimensionality of scRNA-seq, cells with ambiguous identities are artificially assigned to subpopulations, leading to weakened signals and false classifications. Our framework models and tests expression levels of cells with respect to their estimated subpopulations (Fig. 1). The jackstraw strategy accounts for overfitting characteristics of unsupervised clustering. Beyond *P*-values, an empirical Bayes approach is used to derive a probability that a cell truly belongs to an estimated subpopulation, which we call a PIP. This connects an unsupervised classification of high-dimensional data and a fundamental hypothesis framework in a statistically rigorous manner.

Operating characteristics of the proposed methods are demonstrated through comprehensive simulation studies. Three scRNA-seq data analyses are presented using (i) a mixture of Jurkat and 293T cell lines (Zheng *et al.*, 2017), (ii) a Cell Hashing data of peripheral blood mononuclear cells (PBMCs) from eight independent donors (Stoeckius *et al.*, 2018) and (iii) immune populations in 68 579 PBMCs from a single donor (Zheng *et al.*, 2017). The reference implementation (https://CRAN.R-project.org/package=jackstraw) includes *K*-means clustering, partitioning around medoids (PAM; Kaufman and Rousseeuw, 1987) and mini batch *K*-means (MBKM) (Sculley, 2010), which are fast, robust and scalable to millions of single cells.

## 2 Statistical models and methods

Unsupervised clustering of *m* single cells into *K* subpopulations provides *m* cell identities. By modeling *m* cells with respect to their assigned subpopulations, we aim to evaluate cell identities. The observed data $Y_{\left(m,n\right)}$ contain *m* rows and *n* columns. In scRNA-seq data, we assume that single-cell samples are arranged as rows, whereas columns as genomic variables (e.g. genes). A variety of tools (Guo *et al.*, 2015; McCarthy *et al.*, 2017; Qiu *et al.*, 2017; Satija *et al.*, 2015) are used for quality controls and normalization (Fig. 1). Furthermore, dimension reduction may be applied on genomic variables to highlight certain aspects of systematic variations or biomarkers. Therefore, *n* columns may be all available genes, highly variable genes, principal components or others. Nonetheless, when it is clear in context, we simply refer to *n* columns as genes.

Consider that *m* cells form *K* subpopulations, exhibiting distinct systematic patterns of variation. For k=1,…,K, a mutually exclusive subset of cells (*m_k_* out of *m*) are assigned to *k*th cluster. Then, $\sum_{k=1}^{K}m_{k}=m$. Samples within the *k*th cluster exhibit systematic variation that may be summarized by their center, centroid, medoid or other representative $c_{k}(Y)$ for k=1,…K. In *K*-means clustering, the center is defined as the Euclidean mean; the nearest centers (*L*_2_ distance) are then used to classify single-cell samples (Hartigan and Wong, 1979; Lloyd, 1982; MacQueen, 1967). In PAM, the representative mediods are selected from observed samples and *L*_1_ distance is used for membership assignments (Kaufman and Rousseeuw, 1987).

Clusters are viewed as distinct systematic patterns of variation being manifested on subpopulations of cells. Among gene expression profiles of single cells, clusters may reflect cellular heterogeneity. Cells that should be clustered together in a given subpopulation share distinct characteristics that are defined by its center, centroid, medoid or others. Consider there exist unobserved centers **l**_*k*_ and coefficients **b**_*k*_ for k=1,…,K. Then, the data are modeled as:

(1) $$Y_{\left(m,n\right)}=B_{\left(m,K\right)}L_{\left(K,n\right)}+E_{\left(m,n\right)},$$

where E is an independently and identically distributed noise. With respect to a cluster, **b**_*k*_ is consisted of a point mass at zero and a continuous distribution for coefficient values. This spike-and-slab model introduces zero-one latent variable $\gamma_{k}$ with initial inclusion probabilities (George and McCulloch, 1997; Mitchell and Beauchamp, 1988). If a particular *i*th sample is truly associated with **l**_*k*_, $\gamma_{i,k}$ is 1. Otherwise, 0. $b_{k}=\gamma_{k}\beta_{k}$, where $\beta_{k}$ may take on a continuous distribution, quantifying the relationship between L and Y. This allows biological, including cell-to-cell, variation within a cluster. Row-wise means can be easily handled by centering the data.

There have been important developments in unsupervised learning that consider mixture models that improve our understanding and interpretation of data (McLachlan and Peel, 2004; Yeung *et al.*, 2001). However, even model-based clustering does not provide cluster centers and membership assignments that can be used again with the observed data. Our approach learns and incorporates inevitable uncertainty in assigning single-cell samples to clusters that are directly derived from the same set of samples.

### 2.1 Jackstraw test for cluster membership

We propose to use the *F*-statistics to relate the single-cell samples **Y** and the cluster centers $c_{k}(Y)$ for k=1,…,K. Generally, the gene expression profiles of a given single cell **y**_*i*_ can be modeled with the cluster centers $c_{k}(Y)$ and other covariates **X**_*i*_, resulting in an unrestricted full model $y_{i}∼f_{\text{full}}(c_{k}(Y),X_{i})$. Alternatively, a restricted null model provides no information about $c_{k}(Y)$ such that $y_{i}∼f_{\text{null}}(X_{i})$. Then, an unadjusted residual sum of squares measures the discrepancy between **y**_*i*_ and two competing models,

(2) $${\text{RSS}}_{\text{full},i}=\sum (y_{i}-f_{\text{full}}(c_{k}(Y),X_{i}){)}^{2}$$

(3) $${\text{RSS}}_{\text{null},i}=\sum (y_{i}-f_{\text{null}}(X_{i}){)}^{2}$$

**Fig. 2.**
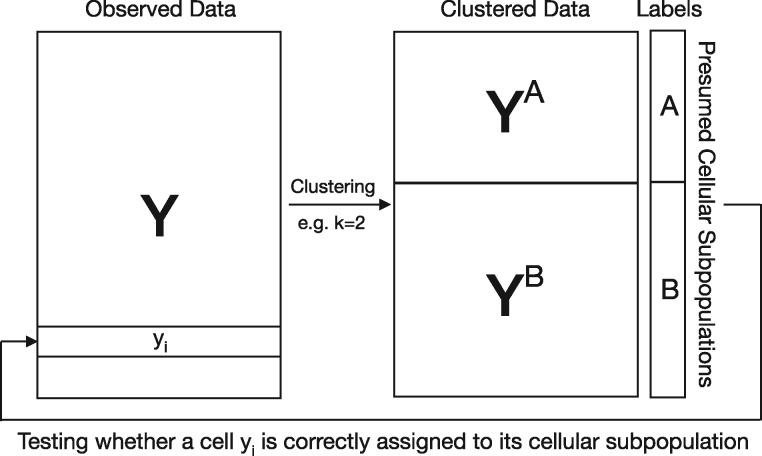
Visual explanation for circular analysis in naively evaluating cluster memberships. In this example, scRNA-seq data are clustered to obtain *K *=* *2 cellular subpopulations. Since cell identities are estimated by clustering scRNA-seq data, testing if a cell is correctly assigned to its presumed cellular subpopulation results in artificially inflated significance. The proposed jackstraw for clustering overcomes this challenge by learning the overfitting inherent in evaluating cluster membership

Then, the unadjusted *F*-statistics for the *i*th single cell is defined as

(4) $$F_{i}=\frac{{\text{RSS}}_{\text{null},i}-{\text{RSS}}_{\text{full},i}}{{\text{RSS}}_{\text{full},i}/(n-p_{\text{full},i})},$$

where $p_{\text{full}}$ denotes the number of parameters in the full model. However, because $c_{k}(Y)$ is estimated from **Y**, there is circular dependency resulting in artificially inflated significance (Fig. 2). To avoid circular analysis, the labels should be an independent variable that is measured externally. Using the data-dependent labels, such as cellular subpopulations derived from clustering, typically fails to control error rates. Therefore, conventional parametric or naive bootstrap-based *F*-tests (Supplementary Material), which expect dependent variables to be modeled by independent variables, are not valid.

We introduce a resampling-based approach to estimate the empirical distribution of *F*-statistics under the null model that adjusts for this circular dependency. This *jackstraw* approach, which was initially developed for PCA and related methods (Chung and Storey, 2015), constructs and utilizes a minimally disruptive *jackstraw data* $Y^{*}$. Out of *m* observed samples, a relatively small number (*s *<* m*) of samples are resampled with replacement, which we call synthetic null samples. Other *m* − *s* observed samples are unchanged. The jackstraw data $Y^{*}$ combines *s* synthetic null samples and intact *m* − *s* observed samples. The cluster structure with *K* subpopulations are preserved in the jackstraw data, as *s* samples became independent and identically distributed (i.i.d.) due to resampling with replacement.

When the jackstraw data are clustered, cluster centers $c_{k}^{*}$($Y^{*}$) are almost identical to the original cluster centers $c_{k}(Y)$ for k=1,…,K (Supplementary Fig. S2). Because of the nature of clustering algorithms, all samples in $Y^{*}$, including *s* synthetic null samples, will be assigned to one of *K* clusters. When a synthetic null sample $y_{i}^{*}$ is assigned to *k*th cluster, an association statistics between $y_{i}^{*}$ and $c_{k}^{*}(Y^{*})$ is under the null model that assumes independence since $y_{i}^{*}$ is i.i.d. by definition. Yet, because $y_{i}^{*}$ is assigned to *k*th cluster, we learn the overfitting characteristics of clustering. Over a large number of iterations b=1,…,B, the empirical distribution of null statistics is formed. This empirical distribution of null statistics is used to evaluate significance of individual samples (Algorithm 1).

Algorithm 1 Jackstraw test for cluster membership

1. Apply the clustering algorithm to the observed data **Y**, resulting in cluster centers **c**_*k*_ for k=1,…,K and membership assignments $b_{i,K}$ for i=1,…,m and K=1,…,k.

2. Compute the observed statistics $F_{1},\ldots ,F_{m}$, where the full models include corresponding cluster centers $c_{k}(Y)$.

3. Create *s* synthetic null samples by resampling with replacement a small proportion of samples s≪m, resulting in a jackstraw data $Y^{*}$, with *m* − *s* observed samples and *s* synthetic null samples.

4. Apply the clustering algorithm to the jackstraw data $Y^{*}$, resulting in cluster centers $c_{k}^{*}(Y^{*})$ and membership assignments $b_{i,K}^{*}$.

5. Compute the null statistics $F_{1}^{*},\ldots ,F_{s}^{*}$, where the full models include corresponding cluster centers $c_{k}^{*}(Y^{*})$.

6. Repeat the above three steps b=1,…,B times to obtain a total s*B of null statistics.

7. Compute the *P*-values by empirically ranking the observed statistics among the null statistics.

The choices of *s* and *B* control the speed of computation, while the total number of null statistics (*s *×* B*) determines the overall *P*-value resolution. For *B* iterations, we need to cluster the jackstraw data *B* times, and for each iteration b=1,…,B, we obtain *s* null statistics. Assuming *s *×* B* is hold constant, a smaller *s* provides more accurate *P*-values, while increasing computational burdens. Therefore, we want to ensure the original clusters are preserved as much as possible, permitting the computational power. As we increase the number of synthetic null samples *s* in $Y^{*}$, the overall systematic variation captured by *K* cluster centers may be increasingly disrupted. Although we use s∼0.1×m for genomic data, the number of clusters (*K*) and the proportion of samples assigned to them ($m_{1},\ldots ,m_{k}$) must be considered.

The reference implementation (https://CRAN.R-project.org/package=jackstraw) uses *K*-means clustering (Hartigan and Wong, 1979; Lloyd, 1982; MacQueen, 1967), PAM (Kaufman and Rousseeuw, 1987) and MBKM (Sculley, 2010). *K*-means clustering is one of the most established and popular algorithms (Hartigan and Wong, 1979; Lloyd, 1982; MacQueen, 1967). Particularly, considering a growing size of scRNA-seq data, *K*-means clustering is orders of magnitude more efficient than hierarchical clustering, graph-based community detection and density-based clustering (Tan *et al.*, 2018; Xu and Su, 2015).

Furthermore, we incorporate a highly scalable mini batch version of *K*-means (Sculley, 2010), where a random subset of single-cell samples are used iteratively to update cluster centers and membership assignments (Steps 1 and 4 in Algorithm 1). Similarly, instead of randomly selecting cluster centers, *K*-means++ initialization may improve its convergence, which is available as a default option in the reference implementation (Arthur and Vassilvitskii, 2007). Because *K*-means clustering relies on Euclidean distance, one may be concerned about its robustness to outliers or generalizability to other distributions. By choosing observed data as cluster centers and using *L*_1_ norm, PAM may perform more appropriately and is included in our jackstraw package.

### 2.2 Posterior inclusion probabilities

When clustering *m* samples into *K* subpopulations, the proposed jackstraw test estimates a probability that an individual cell may have been assigned to a given subpopulation by chance. We further propose to estimate posterior probabilities that *m* cells are correctly assigned to their clusters. This enables probabilistic feature selection and improved visualization of t-SNE, PCA and others.

Consider that the *m* jackstraw *P*-values $p=p_{1},\ldots ,p_{m}$ are obtained for *m* single-cell samples that have been clustered into *K* subpopulations. We estimate a posterior probability that $b_{i}\neq 0$, since non-zero coefficients imply their bona fide inclusion in the clusters:

(5) $$\rho_{i}=\mathrm{Pr}(b_{i}\neq 0|p_{m})$$

(6) $$=1-\mathrm{Pr}(b_{i}=0|p_{m}).$$

PIPs can be readily obtained by estimating $\mathrm{Pr}(b_{i}=0|p_{m})$ through an empirical Bayes approach (Efron, 2007; Efron *et al.*, 2001). In multiple hypothesis testing, $\mathrm{Pr}(b_{i}=0|p_{m})$ is called a local false discovery rate (FDR). With a large amount of samples, it may be advantageous to consider posterior probabilities among each subpopulation or to improve estimation of FDRs and related quantities using prior biological knowledge. There also exist related Bayesian methods that could be explored for specific applications and prior distributions (Barbieri and Berger, 2004; Scott and Berger, 2006).

### 2.3 Feature selection and downstream uses

The proposed methods produce *P*-values and PIPs that are useful in downstream uses. Beyond their statistical properties, they may be used for visualization, feature selection and others.

The proposed *m* PIPs can be flexibly combined for downstream analyses, as to aid feature selection and dimension reduction. When applying the proposed methods to evaluate cell identities in scRNA-seq data, PIPs are used to hard-threshold and soft-threshold single-cell samples. First, in hard-thresholding, cells with low PIPs would be removed or masked for certain downstream analyses, achieving feature selection. For example, a subset of samples above a certain PIP threshold (e.g. > 0.8) may be visualized in reduced dimensions of t-SNE or PCA. Second, in soft-thresholding, PIPs may be used as weights for single cells for downstream analyses. In visualization, one may use PIPs to automatically control transparencies or colors that would emphasize samples with high PIPs. Our single-cell analyses demonstrate these downstream usages.

To select a threshold, one may estimate the proportion of null samples (*π*_0_; Storey and Tibshirani, 2003). Then, samples with high PIPs or small *P*-values above that region would be selected accordingly. This automated procedure is used in the comparison of the proposed jackstraw to feature selection methods (Supplementary Material). Furthermore, we anticipate potential uses in weighted regression or weighted PCA in which cells with large PIPs may be considered more important than those with low PIPs. It may improve a wide range of clustering, such as improved assignments of single-cell samples to subpopulations and regularization of cluster centers.

## 3 Simulation studies

To demonstrate the operating characteristics of the proposed statistical tests, we conducted a comprehensive set of simulation studies, which enabled critical assessment of *P*-values using the ground truth. First, we generated a dataset from the model (Equation 1) while varying an amount of noise ($\sigma^{2}$), a number of cells (*m*) and a number of genes (*n*). Second, we considered a cluster structure from gene expression profiles of 2700 PBMCs. Eight clusters with varying amounts of signals are used to simulate the data. Third, we conducted a Splatter experiment (Zappia *et al.*, 2017) using human-induced pluripotent stem cell (iPSC) lines, in which all cells are derived from *K *=* *3 subpopulations. Last, feature selection methods are applied and compared for cluster membership in scRNA-seq data.

First, we generated a large number of simulation configurations that may reflect scRNA-seq analysis. Generally, we investigated the operating characteristics of the proposed methods in simulated data with $\sigma^{2}=5,10,15$, *m *=* *100, 1000, 2000 and *n *=* *100, 1000, 2000 (Supplementary Fig. S1). Here, we focus on one scenario: *m *=* *1000 cells, *n *=* *100 genes, $\sigma^{2}=10$. Centers are drawn from a Normal (*μ*  =  0, $\sigma^{2}=1$) distribution. Relationships between l and samples are given by dichotomous coefficients B where *b_i_* indicates whether **y**_*i*_ is a member of l for i=1,…,m. Last, $E\overset{i.i.d}{∼}$ Normal (0, $\sigma_{b}^{2}$) with $\sigma_{b}^{2}=10$. Increasing $\sigma_{b}^{2}$ brings these two groups closer and makes clustering more difficult (Supplementary Fig. S3). The proposed jackstraw tests were applied for *K*-means clustering, with *s *=* *100 and *B *=* *5000. Theoretically, the null *P*-values corresponding to the null hypotheses (noise-only samples) should form a Uniform(0,1) distribution, which can be evaluated by the Kolmogorov–Smirnov (KS) test. We repeated a given simulation configuration 100 times independently and investigated how 100 KS test *P*-values meet the joint null criterion (Leek and Storey, 2011). Meeting the joint null criterion demonstrates that the proposed methods overcome circular analysis inherent in using cluster centers and membership assignments.

**Fig. 3.**
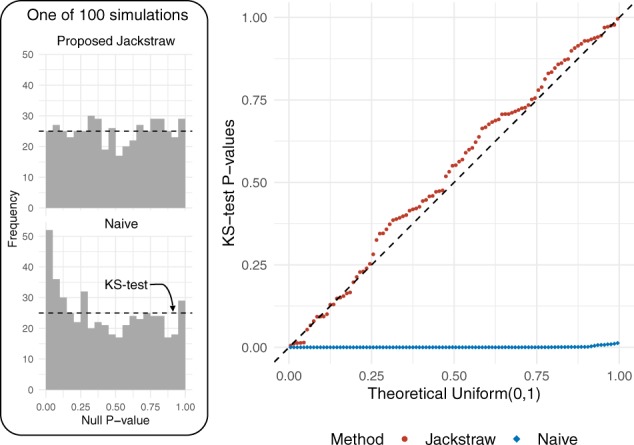
Evaluation of the naive and proposed jackstraw methods for cluster memberships using simulation with *m *=* *1000 cells, *n *=* *100 genes and *σ*  =  10. On the left, null *P*-values (corresponding to null hypotheses) are shown where a left skewed histogram of naive methods demonstrates an anti-conservative bias. In each of 100 simulation studies, null *P*-values are tested for uniformity by one-sided KS test. Then, 100 KS test *P*-values are plotted against a Uniform(0,1) distribution in a QQ plot, where a downward deviation from diagonal dashed line indicates an overall anti-conservative behavior

One hundred KS test *P*-values, estimated from both the jackstraw and conventional *F*-test methods, are visualized against a Uniform(0,1) distribution (Fig. 3). The jackstraw tests satisfy the joint null criterion (Leek and Storey, 2011), where the joint behavior of 100 KS test *P*-values follows an i.i.d. Uniform(0,1) distribution (double KS test *P*-value =0.78). In contrast, the conventional methods are strongly anti-conservative, where 100 KS test *P*-values are strongly skewed toward 0 (double KS test *P*-values $<2.2\times 10^{-16}$). These behaviors are similarly confirmed by additional simulation configurations. We carried out simulation studies by changing $\sigma^{2}=5$ and 15 while keeping *m* and *n* constant (Supplementary Fig. S4). As the clusters become more overlapping with an increase in $\sigma^{2}$, PIPs tend to be smaller (Supplementary Fig. S5). In other words, the distinctiveness of clusters in data is reflected on PIPs. We further changed dimensions *m *=* *100, 1000, 2000 and *n *=* *100, 1000, 2000 in simulation for further confirmation (Supplementary Fig. S6).

Second, we used a dataset of 2700 PBMCs, called pbmc3k from 10× Genomics (https://s3-us-west-2.amazonaws.com/10x.files/samples/cell/pbmc3k/pbmc3k_filtered_gene_bc_matrices.tar.gz) to generate scRNA-seq characteristics. Genes expressed in ≥3 cells and cells with ≥200 non-zero expression values are retained. After removing outliers, we log-normalized the data and regressed out technical variations due a number of unique molecular identifiers (UMIs) and a percentage of mitochondrial gene expression. Among 2638 PBMC samples, we selected 1838 highly variable genes. *K*-means clustering is applied on the resulting 2638 PBMC samples containing 1838 genes, using *K *=* *8. These eight clusters contain 346, 290, 177, 16, 186, 33, 1134 and 456 samples with diverse cluster centers. We use these eight clusters of pbmc3k data and their corresponding numbers of members to generate an identically sized dataset with 10% of i.i.d. null samples. Essentially, we simulated the PBMC dataset, where null samples are known. The proposed method was applied to evaluate cluster membership, with *s *=* *264 and *B *=* *100 (Supplementary Fig. S7). The jackstraw *P*-values corresponding to null samples follow a theoretically correct diagonal line with a KS *P*-value of 0.88. As expected, the true members of clusters correspond to highly significant *P*-values that are skewed toward to 0 (double KS test *P*-values $<2.2\times 10^{-16}$).

Third, we investigated the operating characteristics of the proposed methods when scRNA-seq data are simulated by Splatter (Zappia *et al.*, 2017; Supplementary Material). Using Splatter, we investigated how the proposed jackstraw method operates when all of cells are indeed derived from in *K *=* *3 subpopulations. The parameters for a Splat models are estimated from scRNA-seq data on human iPSC lines from the single-cell Fluidigm C1 platform (Tung *et al.*, 2017). Following the application in Zappia *et al.* (2017), *m *=* *400 cells from *K *=* *3 subpopulations are simulated from probabilities of 0.60, 0.25 and 0.15. We applied the jackstraw for *K*-means clustering on a range of *d* eigenvectors as inspired by SC3 (Kiselev *et al.*, 2017), which resulted in four cases using d=0.04,0.05,0.06 and 0.07m. We found that the *P*-values are highly significant such that almost all of cells are estimated to be included in their subpopulations with $\hat{\pi_{0}}∼0$ (Supplementary Fig. S8).

Fourth, feature selection algorithms are compared using the main simulation scenario (*m *=* *1000 cells, *n *=* *100 genes and $\sigma^{2}=10$). In particular, least absolute shrinkage and selection operator (lasso) (Tibshirani, 1996), elastic net (Zou and Hastie, 2005), max–min parents and children (Tsamardinos *et al.*, 2003) and forward–backward selection with early dropping (Borboudakis and Tsamardinos, 2019) are applied with cross-validation for choosing hyper-parameters (Supplementary Material). For the jackstraw to automatically choose cells (e.g. ‘features’), the resulting *P*-values are used to estimate *π*_0_ (Storey and Tibshirani, 2003) and thresholded accordingly. The total number of positives, the false negative rates (FNRs) and the false positive rates are measured (Supplementary Fig. S8). Generally, the proposed jackstraw method outperforms, by identifying a far greater number of positives at a much lower FNR (Supplementary Fig. S9b). This is expected as most of feature selection algorithms remove correlated features, which is ill-suited for our goal of evaluating cluster membership. The jackstraw methods for cluster membership are designed to take account for the fact that cluster centers are linear combinations of expression profiles.

## 4 Single-cell analyses

Recent scRNA-seq studies obtain gene expression from single cells, in order to elucidate transcriptional heterogeneity (Jaitin *et al.*, 2014; Macosko *et al.*, 2015; Zheng *et al.*, 2017). Cell identities are unknown at a single-cell level, even though heterogeneity is manifested on gene expression. Although cell identities are routinely obtained from unsupervised clustering, it may be important to test if cluster membership (e.g. placing a cell to a particular subpopulation) is correctly inferred. We applied the proposed methods on three scRNA-seq datasets.

Please note that there are a number of analytic steps prior to applying clustering to identify cellular subpopulations (Fig. 1). For example, normalization, gene selection and dimension reduction are considered to account for unwanted technical variation, to overcome a computationally bottleneck and to accentuate biological signals of interest (Brennecke *et al.*, 2013; Hicks *et al.*, 2018; Stegle *et al.*, 2015). This series of challenges require understanding of study designs and goals, exploratory data analysis and sound statistical approaches. Our analyses directly utilize a number of carefully chosen choices in the original analyses.

### 4.1 Mixture of Jurkat and 293T cell lines

**Fig. 4.**
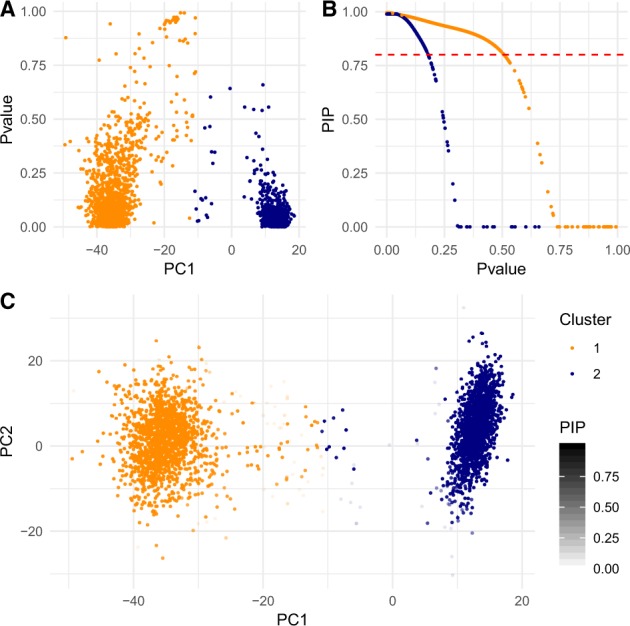
Cell identities in the Jurkat: 293T cell mixture data from Zheng *et al.* (2017). Two distinct cell lines form *K *=* *2 cellular subpopulations. The proposed jackstraw method is applied on the top 10 PCs of UMIs. (**A**) *P*-values from the proposed methods are plotted against the first PC. Two colored points correspond to two clusters. (**B**) At PIP < 0.80, 3.4% of 3381 single cells would be removed. Removing or down-weighting cells with low PIPs serve as feature selection for those with substantial association with presumed cellular subpopulations. (**C**) PIPs control transparency levels on the PC scatterplot. When PIP = 0, the data point is completely transparent

Cells from a mixture of Jurkat and 293T cell lines (50:50) were sequenced using GemCode by 10× Genomics (Zheng *et al.*, 2017). Jurkat and 293T cell lines are highly distinct, being derived from male and female individuals, respectively. Zheng *et al.* (2017) applied *K*-means clustering that separates *m *=* *3381 cells into *K *=* *2 subpopulations. Following quality control, normalization, gene selection and dimension reduction in the original analysis (Zheng *et al.*, 2017), we tested whether individual cells are correctly assigned to one of two subpopulations based on the top 10 PCs of UMI. The proposed jackstraw tests for those clusters were conducted with s=.1×m and *B *=* *1000. *P*-values capture deviation away from two centers, along the first PC axis (Fig. 4a). At PIP < 0.80 (equivalent to 20% local FDRs), 5.97% of 3381 single cells are identified as ambiguous and removed from corresponding clusters (Fig. 4b). We visualized PIPs as levels of transparency in a scatterplot of the top two PCs (Fig. 4c).

Given that a large number of single cells are automatically captured and profiled by a droplet-based platform GemCode, it is known that a single droplet might contain two or more single cells. Known as doublets or multiplets, they may induce biologically irrelevant gene expression profiles in scRNA-seq studies. Through single nucleotide variant detection, Zheng *et al.* (2017) inferred a 3.1% multiplet rate for this 50:50 mixture experiment (Zheng *et al.*, 2017). Furthermore, this error rate approximately linearly increases with the recovered cell number, such that ∼10 000 cells result in >8% multiplet rates (Zheng *et al.*, 2017). Contaminations by multiplets are ubiquitous in high-throughput scRNA-seq platforms (Andrews and Hemberg, 2018). The ambiguous identities of single cells due to multiplets and other source variations would become increasingly challenging as scRNA-seq becomes more high-throughput.

Detection algorithms for multiplets have been developed by simulating artificial multiplets from cellular subpopulations and comparing the observed samples to artificial multiplets (McGinnis *et al.*, 2019). We applied DoubletFinder (McGinnis *et al.*, 2019) on this mixture of cell lines, in which a number of putative doublets are specified by nExp=1,3,5% (details in Supplementary Material). The putative nulls with respect to subpopulations, identified by the jackstraw, significantly overlap with the putative doublets (mean Jaccard index $\bar{J}=0.34$ and *P*-value $<2.2\times 10^{-16}$; Chung *et al.*, 2019). However, stemming from their distinct goals, their assumptions and operating characteristics are distinct (Supplementary Fig. S10). Generally, the proposed jackstraw test for cluster membership complements these multiplet detection methods, which rely on accurate estimation of cellular subpopulations.

### 4.2 Cell hashing and HTODemux classifications

Cell Hashing uses oligonucleotide-tagged antibodies against surface proteins to label single cells (Stoeckius *et al.*, 2018). These labeled single cells can be pooled and sequenced together. The barcoded antibodies which correspond to different origins of cells are used to demultiplex the pooled samples, robustly identifying cell identities. In Stoeckius *et al.* (2018), PBMCs from eight donors were multiplexed in a single run of scRNA-seq. This Cell Hashing resulted in sequencing data of RNAs and hashtag oligonucleotides (HTOs) that are used to classify cells. Normalization and scaling were carried out as suggested in a corresponding Seurat vignette (Butler *et al.*, 2018; Satija *et al.*, 2015). The HTODemux algorithm in Seurat was applied for sample demultiplexing, which provide HTODemux classifications. ‘HTO-A’ through ‘HTO-H’ correspond to eight donors (i.e. ‘Singlet’).

**Fig. 5.**
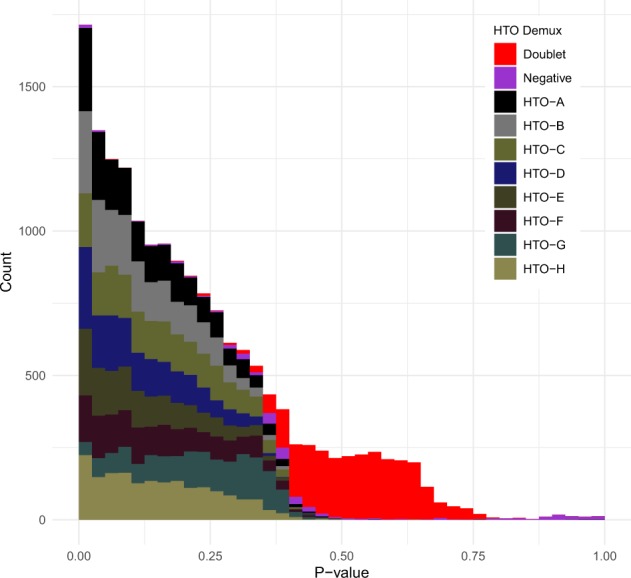
Jackstraw analysis of Cell Hashing data with HTO Demux (Stoeckius *et al.*, 2018). Eight donors provided PBMCs, which are pooled and sequenced together using Cell Hashing (Stoeckius *et al.*, 2018). Identities of 16 916 single cells are estimated by applying the HTODemux algorithm in Seurat. HTO-A thru HTO-H are singlets, corresponding to eight donors. Independently, the proposed method is applied on HTOs, resulting in 16 916 *P*-values for individual cells. The jackstraw *P*-values are in strong agreement with HTODemux classifications

Independent of the HTODemux classifications, the proposed methods were applied on 16 916 single cells in HTO data. The jackstraw estimates *P*-values of association between single cells and their clusters corresponding to different donors. We found that the resulting *P*-values are highly concordant with the HTODemux classifications. The distributions of *P*-values stratified by their classifications are distinct, where ‘Singlet (HTO-*)’ cells are highly significant (Fig. 5). The mean *P*-values corresponding to ‘Singlet (HTO-*)’, ‘Doublet’ and ‘Negative’ were 0.15, 0.53 and 0.49, respectively. Overall, Cell Hashing enables pooling different samples. Application of the jackstraw on the HTO data shows strong agreement with HTODemux classifications (Fig. 5). As Cell Hashing reports statistical errors such as FNR of ∼0.9% (Stoeckius *et al.*, 2018), the jackstraw *P*-values and PIPs may help demultiplexing and control overall error rates.

### 4.3 Immune populations among 68K PBMCs

We analyzed gene expression profiles of PBMCs from a single healthy donor (Zheng *et al.*, 2017). PBMCs in human are consisted of heterogeneous cell types, such as lymphocytes (T, B and NK cells), monocytes and dendritic cells. The original analysis used *K *=* *10 clusters to characterize transcriptional heterogeneity in this 68K PBMC dataset. Our methods identify the most relevant samples for these 10 clusters. Genes that are expressed in >1% of observed cells and single-cell samples with ≥500 genes were retained and processed using Seurat (Butler *et al.*, 2018; Satija *et al.*, 2015). We applied a log-normalization, followed by regressing out technical variations due to batch effects (eight channels), % mitochondrial genes and numbers of UMIs. Directly reflecting the analytical choices in (Zheng *et al.*, 2017), we selected the 1000 most variable genes by their dispersion among 40 507 PBMCs and obtained the top 50 PCs.

**Fig. 6.**
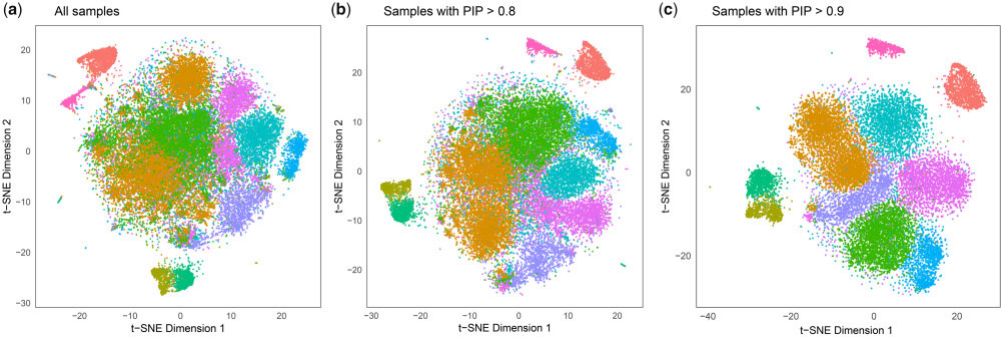
t-SNE projection of PBMCs with the proposed feature selection. Following Zheng *et al.* (2017), 40 507 out of 68 579 PBMCs are retained and the top 50 PCs from 1000 most variable genes are obtained. After normalization and scaling by Seurat, MBKM clustering is applied for *K *=* *10 clusters. The jackstraw method estimated PIPs for 40 537 cells. Thresholding PIPs enable feature selection of single cells that are robust members of presumed cellular subpopulations. (**a**) t-SNE projection using all 40 507 PBMCs. (**b**) 34 134 samples with PIPs > 0.8 and (**c**) 22 407 samples with PIPs > 0.9. Colors correspond to 10 clusters

We applied MBKM clustering (Sculley, 2010) on the top 50 PCs obtained from this PBMC data. The proposed jackstraw test for cluster membership was applied with 10% synthetic null samples and 100 iterations (Supplementary Fig. S12). The proportion of null samples is estimated to be $\hat{\pi_{0}}=0.124$. At PIP > 0.80 and > 0.90, we found that 34 134 (84.2%) and 22 407 (55.3%) single-cell samples are assigned to their corresponding 10 clusters, respectively. Using a perplexity parameter of 30, t-SNE projection after our feature selection suggests that the proposed methods help remove cells with ambiguous identities (Fig. 6). Due to a stochastic nature of t-SNE, separate runs may result in different projections. Therefore, one may also remove a subset of cells with low PIPs using the original t-SNE projection, which is shown in Supplementary Figure S13.

Note that with 100% of samples for initialization and 10% batch size, MBKM clustering took 3–4 s for 10 starts and 1000 maximum iterations. In contrast, *K*-means clustering on this dataset required 20–21 s (MacBookPro i5 2.4 GHz).

## 5 Discussion

ScRNA-seq enables genome-wide quantification of gene expression in tens of thousands of single cells. Transcriptional heterogeneity in scRNA-seq data is routinely characterized by estimating cell identities using unsupervised clustering. We introduce a set of methods to rigorously test clustering-based cell identities, estimate PIPs and improve downstream visualization. By learning the overfitting characteristics inherent in applying clustering to high-dimensional data, the proposed methods guard against artificially inflated significances.

Our key insight is to generate and re-cluster the jackstraw data, in which a small number of synthetic null samples are used to derive the empirical null distribution. Comprehensive simulation studies demonstrated accurate operating characteristics, including rigorous error controls. Applications on three scRNA-seq datasets showcase how the proposed methods enable probabilistic feature selection and improved projections of PCA or t-SNE. Interestingly, ambiguous single cells such as multiplets are shown to contaminate high-throughput scRNA-seq data. Therefore, the proposed methods may help in quality control and identification of major molecular signatures.

When any clustering method is applied for estimation of single-cell identities, a number of clusters must be determined. Identifying an optimal number of clusters is a fundamental challenge (Akaike, 1974; Bock, 1985; Jain and Moreau, 1987; Tibshirani *et al.*, 2001) that is beyond the scope of this study. Even if a clustering algorithm sidesteps an explicit input for a number of clusters, hyper-parameters such as a resolution, a number of nearest neighbors or a modularity are required from the user. These hyper-parameters indirectly set the number of clusters for a given scRNA-seq data. Overall, exploration of data with domain knowledge and computational analysis would help finding these parameters.

The proposed methods can aid in feature selection, biomarker identification and visualization. First, cells with low PIPs may be removed from downstream analyses, in a similar manner to quality control. Second, PIPs may be used as visual elements (e.g. alpha levels) in scatter plots and others. Third, using PIPs, one may potentially carry out weighted regression or weighted PCA. Fourth, cells with high PIPs may be used for identifying genes that are differentially expressed across conditions or labels. On the other hand, some scRNA-seq data may contain a multi-level structure. This resulted in iteratively applying a clustering algorithm, often supported by qualitative analysis and biological expertise (Macosko *et al.*, 2015; Zheng *et al.*, 2017). Such multi-level clustering may be improved if the proposed PIPs and feature selection are applied. In the future, the jackstraw may be further developed into an integrated method for Bayesian multilevel clustering.

The jackstraw tests for latent variables (Chung and Storey, 2015) have been used in a variety of genomic studies (Chung *et al.*, 2017; Farré *et al.*, 2015; Macosko *et al.*, 2015; Zheng *et al.*, 2017). Complementing this, the proposed tests help evaluate cluster membership, such that clustering-based subpopulations can be rigorously used in downstream analyses. This opens new possibilities for selecting canonical cluster members, shrinking cluster centers and guiding the choice of stable clusters. Because the proposed methods are not limited to scRNA-seq, we anticipate its adaptation in other data-intensive fields.

## Supplementary Material

btaa087_Supplementary_Data
