# Supplementary material for: Statistical significance of cluster membership for unsupervised evaluation of cell identities
Source: Bioinformatics. 2020 Mar 6;36(10):3107–14. doi: 10.1093/bioinformatics/btaa087 (PMC7214036; doi:10.1093/bioinformatics/btaa087)
Supplement: btaa087_Supplementary_Data [file btaa087_supplementary_data.pdf]

Supplementary Information

Statistical significance of cluster membership for unsupervised evaluation of single cell identities

Neo Christopher Chung

1 Splat Simulation Study

There are simulation frameworks for gene expression profiles from scRNA-seq (Vallejos *et al.*, 2015; Korthauer *et al.*, 2016; Zappia *et al.*, 2017). However, most do not allow specification of cluster memberships or inclusion of null gene expression profiles. Particularly, BASiCS (Bayesian Analysis of Single-Cell Sequencing) (Vallejos *et al.*, 2015) and scDD (single cell Differential Distribution) (Vallejos *et al.*, 2015) are concerned with differential expression or differential distribution of genes. Many consider subpopulations that are defined a prior, e.g., case-control studies and known tissue types. Their simulation frameworks were not suitable for our unsupervised studies of scRNA-seq data where cell identities are unknown and estimated from clustering.

Alternatively, using Splat (Zappia *et al.*, 2017) we conducted a simulation study in which all of cells belong to their subpopulations. Using `Splatter` package, the simulation parameters are estimated from scRNA-seq data on human induced pluripotent stem cell (iPSC) lines (Tung *et al.*, 2017). Then, following the original clustering application (Zappia *et al.*, 2017), $m = 400$ single cells from $K = 3$ subpopulations are simulated from probabilities of 0.60, 0.25, and 0.15. Note that in these simulated data, the ground truth is that those cells indeed are related to one of $K = 3$ subpopulations. Being blind to simulation parameters, we applied the jackstraw for K-means clustering on d eigenvectors in a similar manner to its application of SC3 (Kiselev *et al.*, 2017). SC3 sets d to be $4\% - 7\%$ of a number of cells m. Therefore, we demonstrate 4 different scenarios using $d = 0.04 \times 400, 0.05 \times 400, 0.06 \times 400, 0.07 \times 400$. We found that the p-values are highly significant such that most, if not all, of cells are estimated to be included in their subpopulations (Figure S8). π_0 is estimated to be ~ 0 in all of four analyses.

2 Comparison with Feature Selection Methods

The major goal of clustering scRNA-seq data is to identify coherent subpopulations in which cells share similar gene expression patterns. The proposed methods, which produce p-values and posterior inclusion probabilities (PIPs), can be used to filter out cells whose expression profiles are weak or null with respect to estimated subpopulations in scRNA-seq data. P-values or PIPs may be thresholded such that members of clusters with high p-values or low

PIPs are removed. This is somewhat analogous to feature selection in machine learning, where features correspond to cells in this context. In order to automatically select this threshold for the jackstraw, one may estimate and remove the proportion of null features (π_0). There have been a wide range of feature selection methods, which attempt to identify important predictors in high-dimensional settings. Although their comprehensive evaluation for clustering is beyond the scope of this manuscript, we have compared the proposed method to several well-known feature selection methods.

We have considered and compared least absolute shrinkage and selection operator (lasso) (Tibshirani, 1996), elastic net (Zou and Hastie, 2005), Max-Min Parents and Children (MMPC) (Tsamardinos *et al.*, 2003), Forward-Backward selection with Early Dropping (FBED) (Borboudakis and Tsamardinos, 2019). Generally, we used recommended cross-validation and settings given in their implementations. The lasso is one of the most popular feature selection methods used in genomics, which introduces a L_1 penalty on a regression model. We performed 20-fold cross validation to identify an optimal regularization parameter λ_{lasso}. The elastic net improved upon the lasso by introducing an additional L_2 penalty and corresponding λ_{en}. In order to find those hyper-parameters, we ran 20-fold cross validation on λ_{lasso} and $\lambda_{\text{en}} \in [0.05, 0.95]$. MMPC identifies the parents and children of the Bayesian network of observed data. This requires two hyper-parameters; α for statistical significances and κ for conditional independence tests. Due to high computational costs, cross-validation for $\alpha \in [0.01, 0.10]$ and $\kappa \in (1, 50)$ was conducted initially and used for subsequent simulation studies. FBED combines popular forward and backward feature selections with conditional independence tests (Borboudakis and Tsamardinos, 2019). FBED is conducted 10 times using both extended Bayesian Information Criterion (eBIC) and likelihood ratio (LR) tests. They are shown as `FBED_BIC` and `FBED_LR` in Figure S9.

We investigate whether these methods can accurately select features that belong to a given cluster in the main simulation scenario with $m = 1000$, $n = 100$, and $\sigma^2 = 10$. The total number of positives, the false negative rates (FNR), and the false positive rates (FPR) are investigated for 6 different approaches (Figure S9). Note that the total number of true positives is 500. We found that the jackstraw can identify a greater number of positives at a much lower FNR (Figure S9(b)). Generally, other feature selection methods declare a substantially smaller number of cells as positives for a given cluster, as they discriminate against correlated features. Consequently, a small number of positives result in an artificially low FPR (Figure S9(c)). The proposed method can keep FNR substantially low (median = 0.1280), while still maintaining a low median of FPR = 0.028. Note that the goal of other feature selection methods is prediction accuracy, whereas the jackstraw performs statistical inference. These feature selection methods are designed to avoid selecting highly correlated features. This assumption is ill-suited when attempting to identify all features that should be included in a cluster. The jackstraw method for cluster membership is designed to take account for the fact that cluster centers are linear combinations of features.

3 Selecting K using Clustering Validation Methods

For a successful clustering application, there are multiple challenging steps, including but not limited to selecting a number of clusters K, identifying K clusters with their distinct

characteristics, and assigning variables to one of K clusters by some distance/similarity measures. One challenge is to find an optimal number of clusters K, which is often an input into clustering algorithms. Even if hierarchical clustering, community detection, and related algorithms may not require K upfront, it is implicitly set by other hyper-parameters or required later for partitioning. There has been active development of cluster-wide stability measures to help select K. Some of the most popular methods are Rand (Rand, 1971), Dunn (Dunn, 1974), and silhouette (Rousseeuw, 1987) indices. The silhouette index is the average distance between clusters, the Dunn index is a ratio of minimum inter-cluster separation to maximum intra-cluster distance, and the Rand index measures similarity between results from two different clustering. There have also been hypothesis testing approaches for cluster stability (Bock, 1985; Liu *et al.*, 2008; Huang *et al.*, 2015). These formulations quantify how a cluster is homogenous and separated from other clusters. Taken together, these validation methods differ substantially from the proposed method which tests cluster memberships. The proposed p-values or posterior inclusion probabilities (PIP) are estimated for individual members of clusters (e.g., cells), whereas the aforementioned methods are concerned with a cluster-level stability (e.g., a group of cells). Overall, it is important to select appropriate K prior to running the jackstraw for cluster memberships.

4 Detection of Multiplets in scRNA-seq Data

Large-scale single cell RNA-sequencing is enabled by isolating single cells using droplet microfluidics (Macosko *et al.*, 2015; Zheng *et al.*, 2017), nano wells (Cao *et al.*, 2017; Gierahn *et al.*, 2017), and others. Without human supervision, cells are randomly captured into droplets or wells. In such platforms, single cell isolation is not guaranteed, although a low cell concentration input increases a chance of successful single cell isolation. When multiple cells are encapsulated together, they are called multiplets. To identify and remove multiplets analytically, there have been a few algorithms, such as DoubletDecon (DePasquale *et al.*, 2018), DoubletFinder (McGinnis *et al.*, 2019), and Scrublet (Wolock *et al.*, 2019). The key insight among these algorithms is to simulate artificial multiplets from cellular subpopulations. Often taking clusters as presumed cellular subpopulations, artificial multiples are calculated from averaging expression values of randomly selected pairs (or more cells) from distinct clusters. Then, similarities or distances between observed cells and artificial multiplets are measured. Expression profiles that are close to artificial multiplets are putative multiplets.

The proposed jackstraw test for cluster membership complements these multiplet detection methods, which rely on accurate estimation of cellular subpopulations. In addition to applying the jackstraw on the mixture of Jurkat and 293T cell lines, we applied DoubletFinder (McGinnis *et al.*, 2019) for the top 2 PCs. The PC neighborhood size used to compute the proportion of artificial nearest neighbors ($pANN$), pK, was estimated from their parameter sweep algorithm. Using $pN = 0.25$ as recommend, $pK = 0.005$ was found to maximize $BCmvn$, followed by a second peak at $pK = 0.07$. Lastly, $nExp$, which must be defined by the user to threshold $pANN$, is set to $1, 3, 5\%$ (Figure S10). Essentially, if a given cell has many neighbors that are artificial doublets, it is deemed a putative doublet. The putative nulls with respect to subpopulations may sometimes coincide with putative dou-

blets, where the mean Jaccard coefficient $\bar{J} = 0.34$ (Chung *et al.*, 2019). Nonetheless, their assumptions and operating characteristics are distinct. If a large proportion of multiplets are suspected (e.g., due to high cell concentration), multiplet detection algorithms should be applied first.

5 Conventional F-tests

For completeness, we provide how one may apply conventional F-tests for association between the observed data $\mathbf{Y}$ and the cluster centers $\mathbf{c}_k(\mathbf{Y})$. $\mathbf{c}_k(\mathbf{Y})$ estimated from $\mathbf{Y}$ is used as an independent variable in a model. Illustrated in Figure 2, due to obtaining the labels from $\mathbf{Y}$, a conventional regression model results in a circular analysis. Specifically, after $\mathbf{Y}$ are resampled with replacement, their F-statistics with respect to $\mathbf{c}_k(\mathbf{Y})$ are used to form an empirical distribution of null statistics. Observed F-statistics are compared to this distribution to obtain "naive" p-values. This circular analysis inflates statistical significance, since $\mathbf{Y}$ are used twice to estimate the cluster centers $\mathbf{c}_k(\mathbf{Y})$ and to conduct F-tests. Essentially, this represents how the bootstrap or the permutation would be applied to cluster membership, without considering circular dependency (Figure 2). We apply the conventional F-tests in simulation studies to demonstrate how the proposed approach overcomes this type of overfitting.

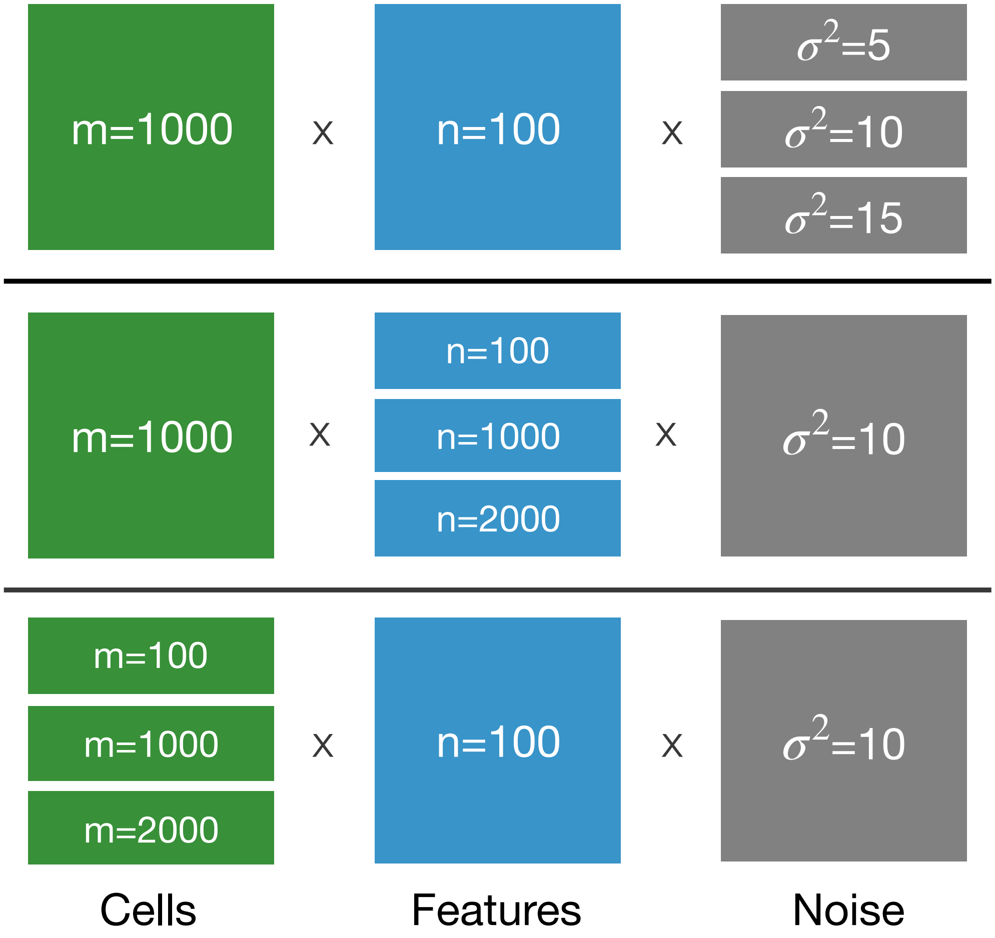

Figure S1: Configurations for simulation studies. We conducted a wide variety of simulation studies with $\sigma^2 = 5, 10, 15$, $m = 100, 1000, 2000$, and $n = 100, 1000, 2000$. First, three simulation configurations where a noise level is varied through σ^2. Second, a number of cells m is varied while keeping a number of features (e.g., genes) $n = 100$. Third, a number of features n is varied while keeping $m = 1000$. Other simulation studies using parameters estimated from scRNA-seq studies are not included in this visual summary.

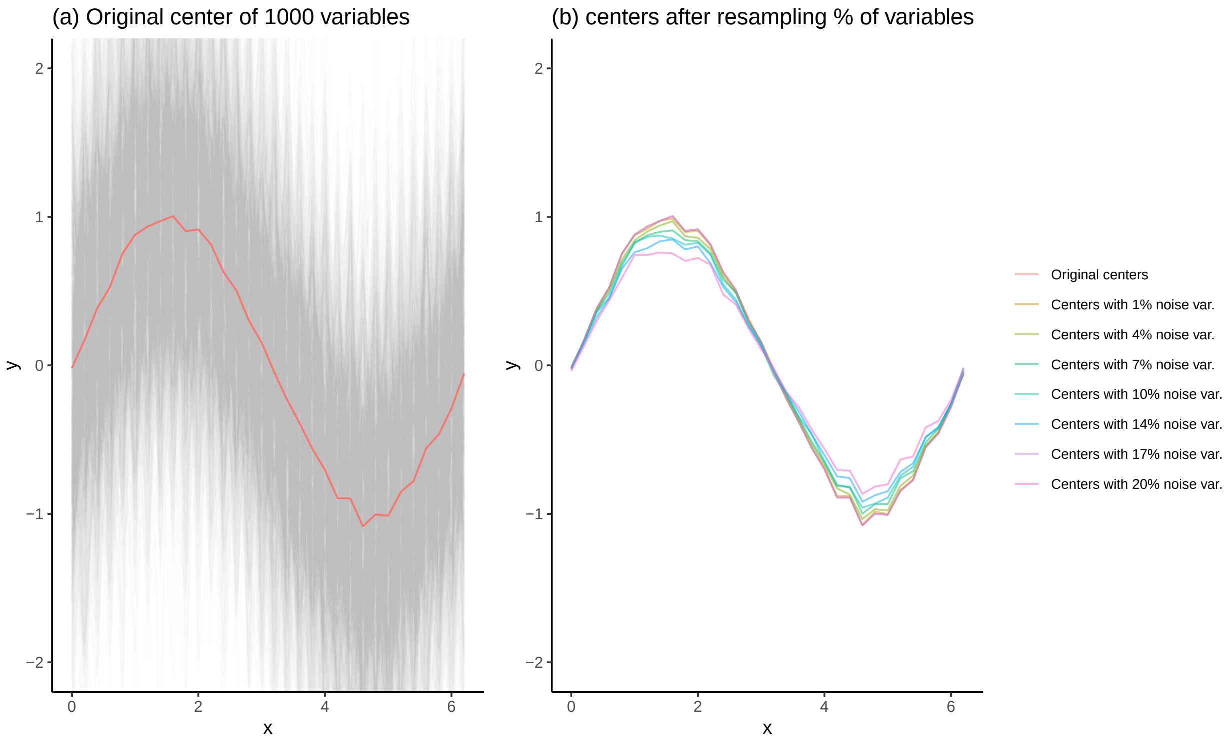

Figure S2: Impact of resampling a small proportion of variables on centers. (a) A total of $m = 1000$ vectors are simulated from a sine function and a noise. (b) A small proportion ($s = 1\% - 20\%$ of m) are resampled with replacement, as done in the jackstraw method. The centers are calculated with added synthetic nulls. They closely approximate the original center (orange line).

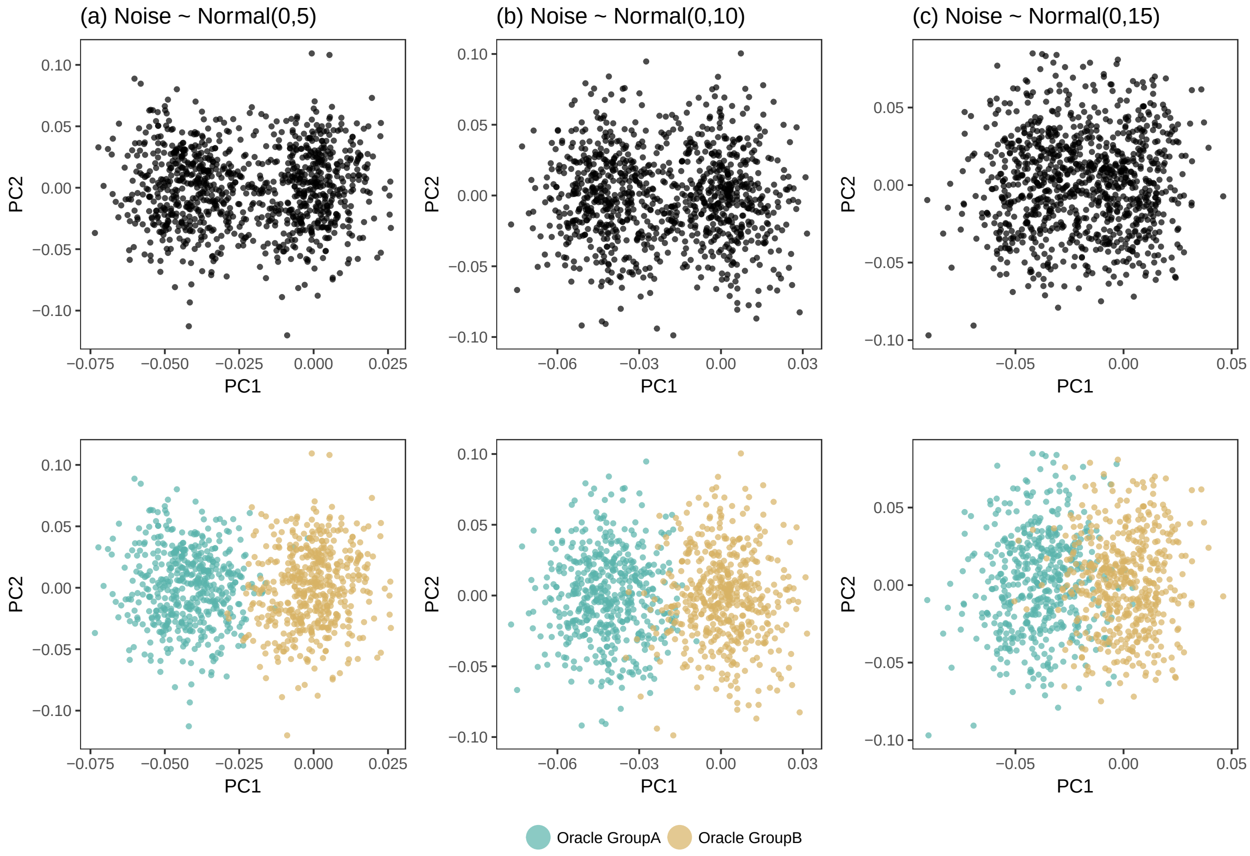

Figure S3: Scatterplots of the top 2 principal components (PCs) from the simulated data. *Oracle Groups* are shown in colors. An increasing level of noise, $\sigma^2 = 5, 10, 15$ brings samples from two different true centers closer together.

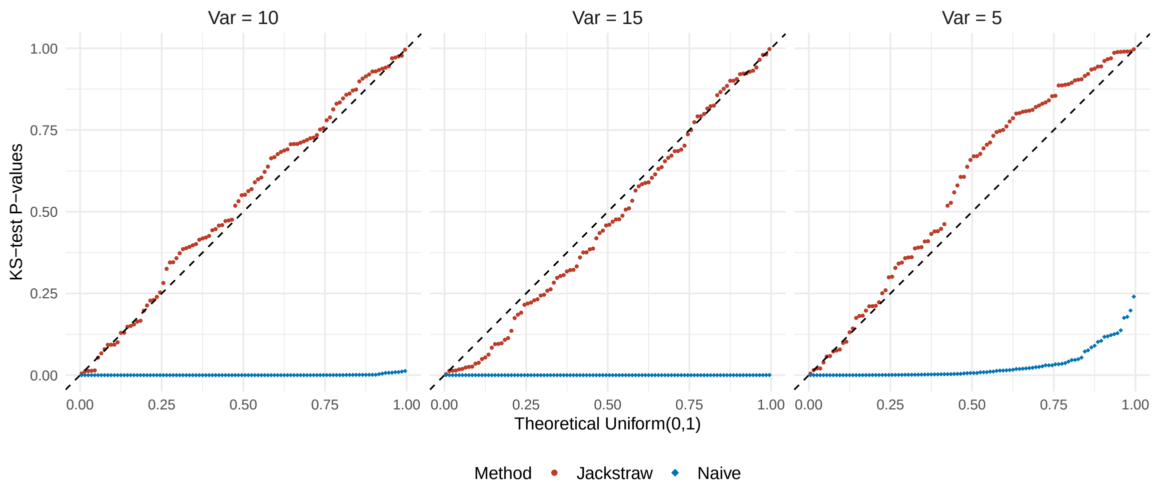

Figure S4: QQ-plots from simulation studies with *(a)* $\sigma^2 = 5$; *(b)* $\sigma^2 = 10$; *(c)* $\sigma^2 = 15$. As shown in Figure S3, an increasing σ^2 results in more overlapping between clusters. Null p-values from the proposed jackstraw and conventional "naive" tests are examined by KS tests in which the anti-conservative behavior is evaluated. Each simulation study results in an one-sided KS-test p-value (see *Simulation Studies* for details). For each of two methods, a total of 100 KS-test p-values are plotted against the Uniform(0,1) distribution. KS test p-values meeting the joint null criterion Leek and Storey (2011) would approximately follow a diagonal line ($\stackrel{i.i.d}{\sim}$ Uniform(0,1)), whereas a downward deviation indicates an anti-conservative bias.

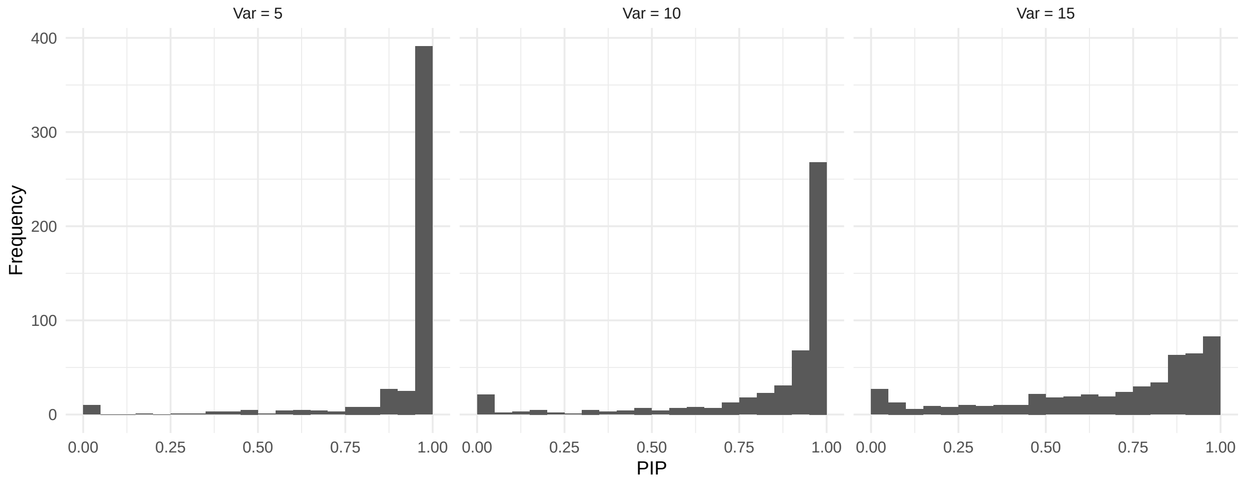

Figure S5: Histogram of PIPs as a function of cluster distinctiveness. As σ^2 increases, the clusters become gradually overlapping as in Figure S3. The jackstraw method for cluster membership is applied in each case, and PIPs related to *Oracle Group A* are visualized in histograms. Other things held constant, PIPs tend to be smaller with an increasing overlap among clusters.

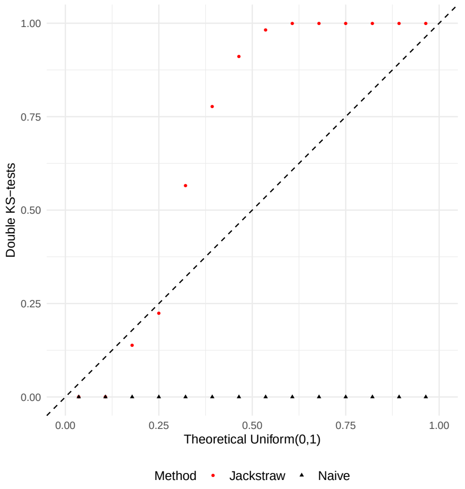

Figure S6: QQ plots of one-sided double KS p-values from simulation configurations in Figure S1. Parameters in simulation studies are varied to generate 7 simulation configurations. Both centering and uncentering simulated data are evaluated. We conducted a total of 14 double KS-tests for anti-conservative bias in the proposed jackstraw and naive methods. A downward deviation from a diagonal line, as evident in the naive method indicates an anti-conservative bias.

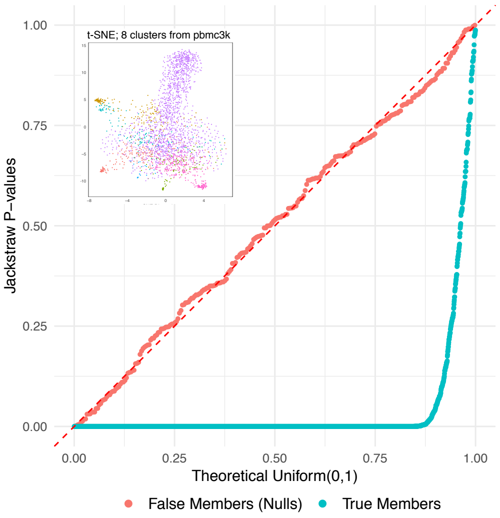

Figure S7: Simulation study using 8 clusters derived from scRNA-seq data. After applying Seurat pre-processing, eight clusters are obtained from a scRNA-seq dataset of 2638 PBMCs from 10X Genomics (available for download as `pbmc3k`). An equal amount of 2638 single cell samples are simulated whose t-SNE projection is shown in an insert. 264 (10%) noise-only samples correspond to null hypotheses, whose p-values should be uniformly distributed. The proposed method was applied on this PBMC-simulated data with $s = 0.1 \times m$ and $B = 1000$. P-values corresponding to null hypotheses should follow a theoretically correct diagonal dashed line.

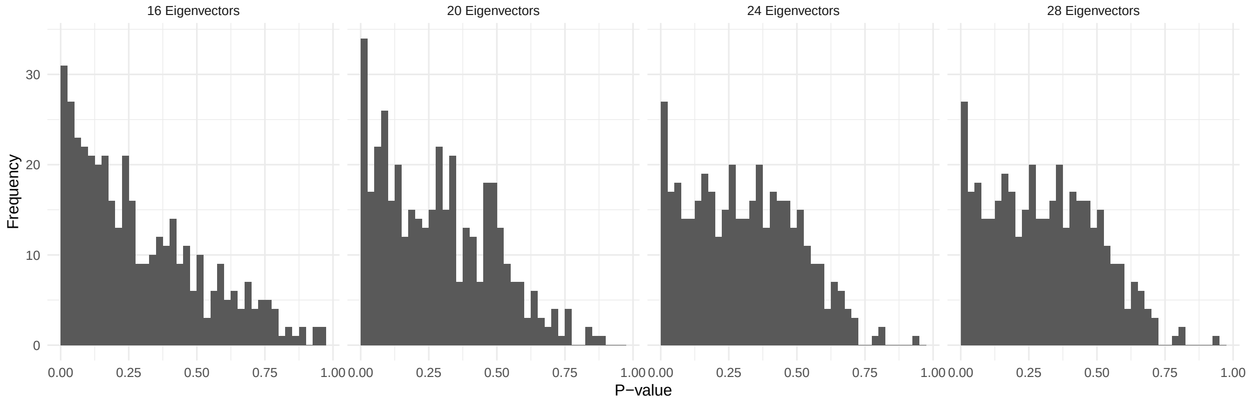

Figure S8: Histograms of jackstraw p-values from Splat-based simulation study (Zappia *et al.*, 2017). Based on scRNA-seq data on human induced pluripotent stem cell (iPSC) (Tung *et al.*, 2017), $m = 400$ cells are simulated from $K = 3$ subpopulations. Following a recommended approach of SC3 Kiselev *et al.* (2017), we applied clustering on d eigenvectors where d ranges $4\% - 7\%$ of a number of cells m. All $m = 400$ cells are truly coming from one of $K = 3$ subpopulations such that highly significant p-values are expected.

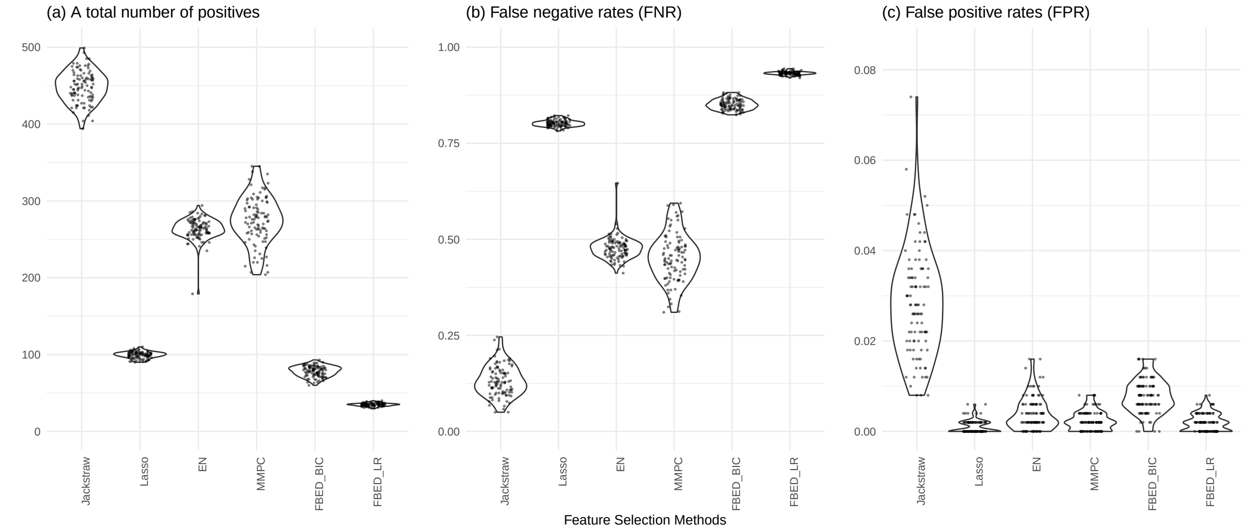

Figure S9: Comparison of the proposed jackstraw method to the feature selection methods. The main simulation scenario with $m = 1000$, $n = 100$, and $\sigma^2 = 10$ is used to evaluate the proposed jackstraw method as a feature selection method, in which π_0 is directly estimated. In comparison, least absolute shrinkage and selection operator (Lasso), elastic net (EN), max-min parents and children (MMPC), and forward backward early dropping with an extended bayesian information criterion (FBED_BIC) and with likelihood ratio tests (FBED_LR) are applied on identical data. Cross validation and recommended implementations are used to select hyper-parameters. This evaluation is carried out 100 times independently, in which (a) a total number of positives declared by each method, (b) false negative rates, and (c) false positive rates are calculated.

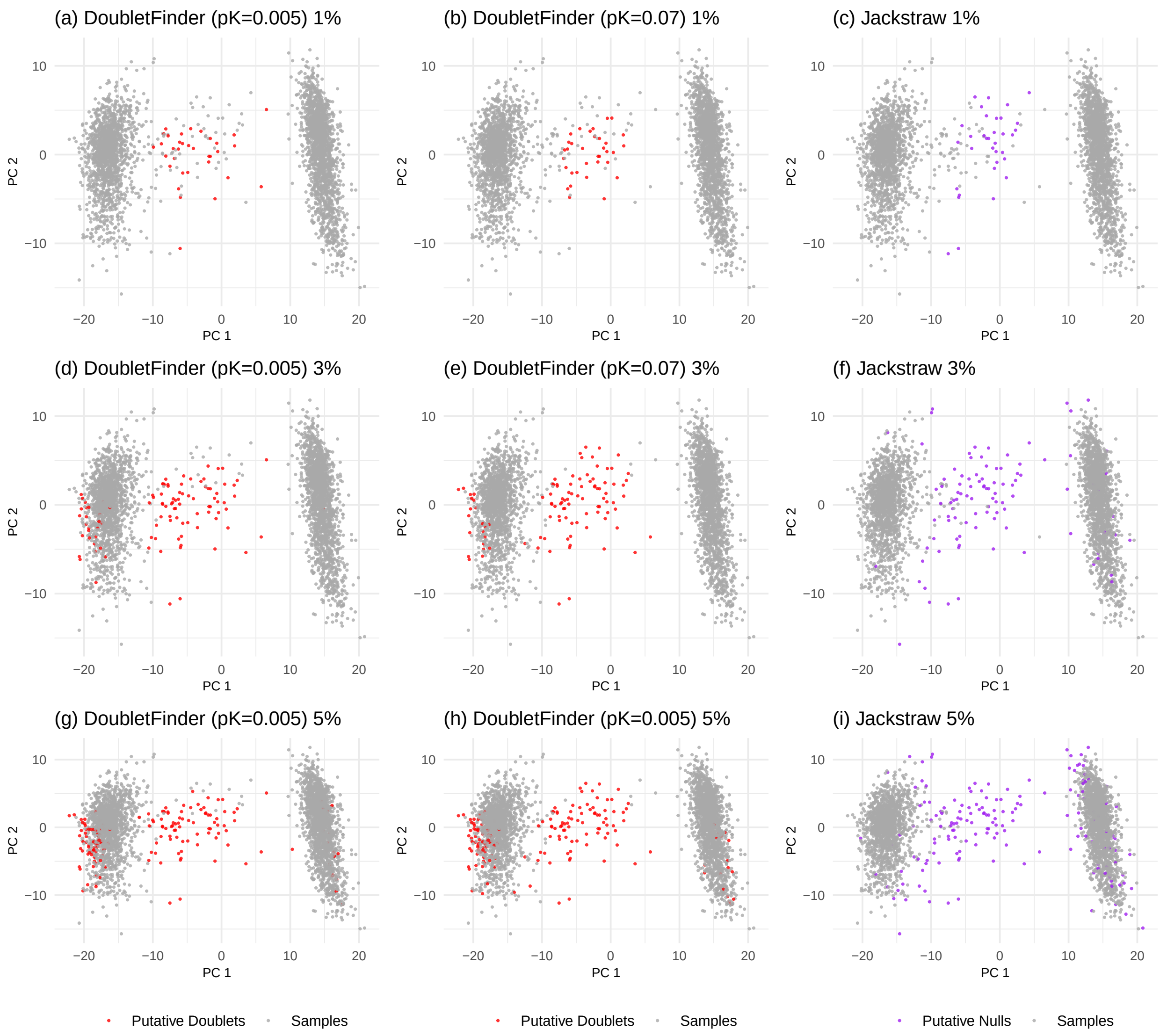

Figure S10: Comparison of the results from applying the jackstraw and DoubletFinder on the mixture of Jurkat and 293T cell lines. DoubletFinder (McGinnis *et al.*, 2019) predicts putative doublets (or multiplets) which are multiple cells erroneously encapsulated together in scRNA-seq platforms. Artificial doublets are simulated by combining gene expression profiles from multiple clusters and are compared against observed expression profiles in reduced dimensions. The PC neighborhood size for DoubletFinder was selected as $pK = 0.005, 0.07$, corresponding to two peaks maximizing $BCmvn$ using parameter sweeps (McGinnis *et al.*, 2019). In contrast, the proposed jackstraw method for clustering identifies cells that do not belong to its estimated subpopulation, which may be called putative nulls. While putative nulls may sometimes coincide with putative doublets ($\bar{J} = 0.34$), their operating characteristics are distinct.

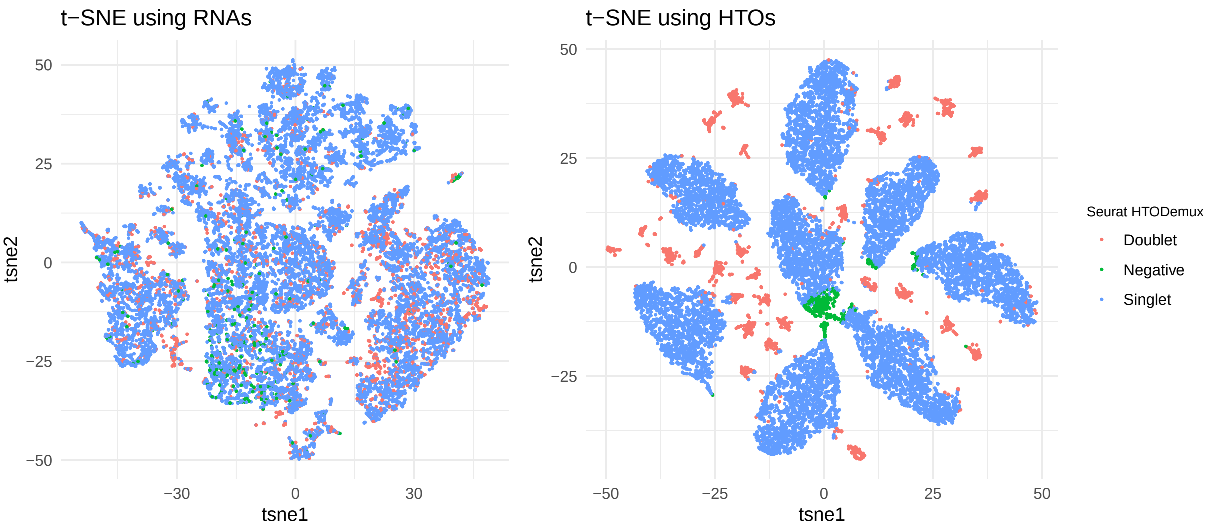

Figure S11: Comparison of RNAs and hashtag-oligos (HTOs) data from the cell hashing study (?). PBMCs from 8 donors were pooled and sequenced together. Cells were labeled with CD45 as a hashing antibody, so that their identities can be extracted from sequencing data of HTOs. The individual points, corresponding to single cells, in t-SNE projections are colored by their classifications based on the Seurat's HTODemux algorithm. Those two datasets contain distinct systematic patterns of variation with divergent t-SNE projections. When K-means clustering is applied on RNA and HTO datasets separately, the overlap was minimal with an adjusted Rand index of 0.0056.

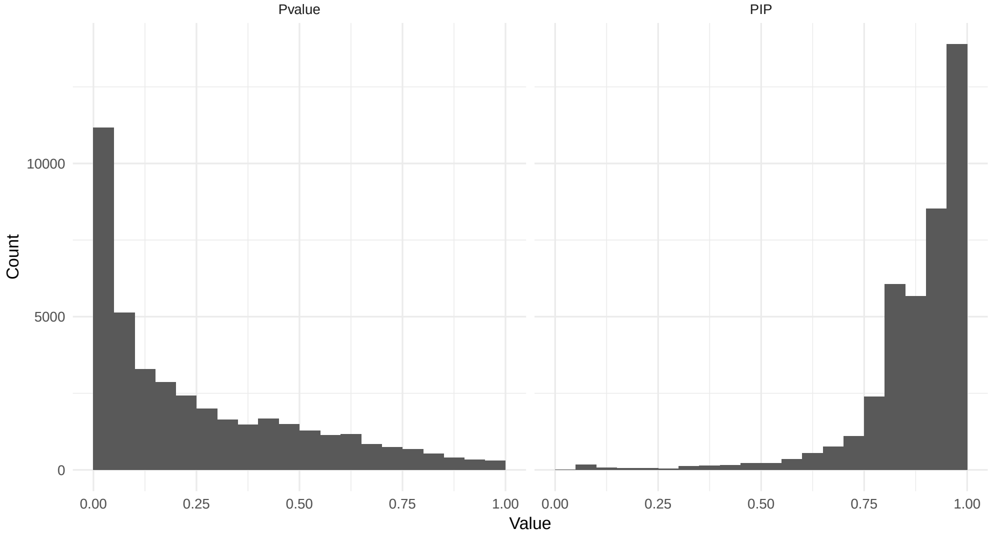

Figure S12: P-value and PIP histograms for applying the proposed methods on 68K PBMC dataset. Mini batch K-means clustering is applied on the top 10 PCs of 1000 variable genes from a processed and normalized 68K PBMC data.

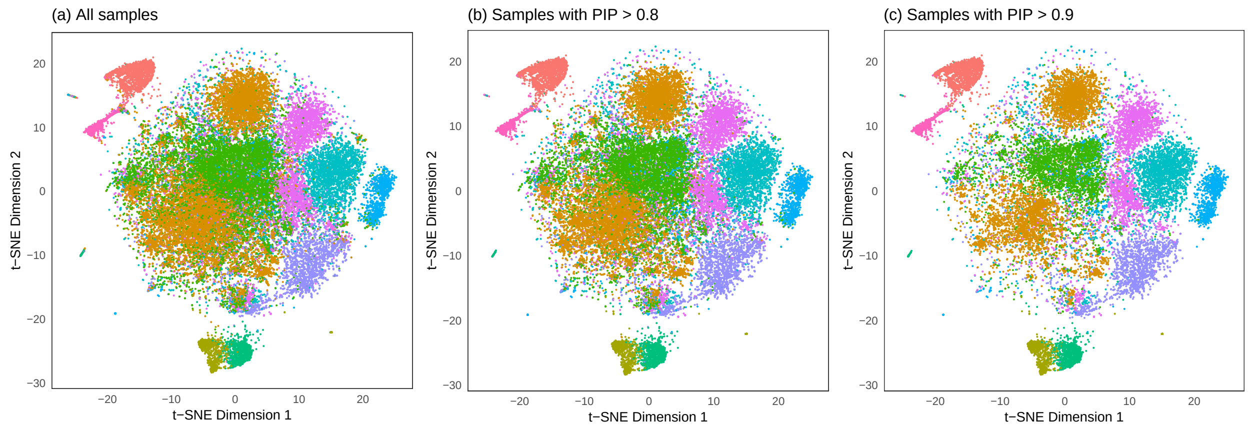

Figure S13: t-SNE projection of PBMCs without recomputing t-SNE. Proposed feature selections for 10 clusters are shown. Instead of recomputing t-SNE, which may stochastically give different projections, the original t-SNE projection using all samples is used in (a)-(c). Then, *(a)* Colors correspond to 10 clusters, which are consistent in three scatterplots.

References

Bock, H.-H. (1985). On some significance tests in cluster analysis. *Journal of classification*, **2**(1), 77–108.

Borboudakis, G. and Tsamardinos, I. (2019). Forward-backward selection with early dropping. *The Journal of Machine Learning Research*, **20**(1), 276–314.

Cao, J., Packer, J. S., Ramani, V., Cusanovich, D. A., Huynh, C., Daza, R., Qiu, X., Lee, C., Furlan, S. N., Steemers, F. J., Adey, A., Waterston, R. H., Trapnell, C., and Shendure, J. (2017). Comprehensive single-cell transcriptional profiling of a multicellular organism. *Science*, **357**(6352), 661–667.

Chung, N. C., Miasojedow, B., Startek, M., and Gambin, A. (2019). Jaccard/tanimoto similarity test and estimation methods for biological presence-absence data. *BMC Bioinformatics*, **20**(S15).

DePasquale, E. A., Schnell, D. J., Valiente-Alandí, Í., Blaxall, B. C., Grimes, H. L., Singh, H., and Salomonis, N. (2018). DoubletDecon: Cell-state aware removal of single-cell RNA-seq doublets. *bioRxiv*, **pre-print**.

Dunn, J. C. (1974). Well-separated clusters and optimal fuzzy partitions. *Journal of Cybernetics*, **4**(1), 95–104.

Gierahn, T. M., Wadsworth, M. H., Hughes, T. K., Bryson, B. D., Butler, A., Satija, R., Fortune, S., Love, J. C., and Shalek, A. K. (2017). Seq-well: portable, low-cost RNA sequencing of single cells at high throughput. *Nature Methods*, **14**(4), 395–398.

Huang, H., Liu, Y., Yuan, M., and Marron, J. (2015). Statistical significance of clustering using soft thresholding. *Journal of Computational and Graphical Statistics*, **24**(4), 975–993.

Kiselev, V. Y., Kirschner, K., Schaub, M. T., Andrews, T., Yiu, A., Chandra, T., Natarajan, K. N., Reik, W., Barahona, M., Green, A. R., and Hemberg, M. (2017). Sc3: consensus clustering of single-cell rna-seq data. *Nature methods*, **14**, 483–486.

Korthauer, K. D., Chu, L.-F., Newton, M. A., Li, Y., Thomson, J., Stewart, R., and Kendziorski, C. (2016). A statistical approach for identifying differential distributions in single-cell RNA-seq experiments. *Genome Biology*, **17**(1).

Leek, J. T. and Storey, J. D. (2011). The joint null criterion for multiple hypothesis tests. *Statistical Applications in Genetics and Molecular Biology*, **10**(1), Article 28.

Liu, Y., Hayes, D. N., Nobel, A., and Marron, J. (2008). Statistical significance of clustering for high-dimension, low-sample size data. *Journal of the American Statistical Association*, **103**(483), 1281–1293.

Macosko, E. Z., Basu, A., Satija, R., Nemesh, J., Shekhar, K., Goldman, M., Tirosh, I., Bialas, A. R., Kamitaki, N., Martersteck, E. M., *et al.* (2015). Highly parallel genome-wide expression profiling of individual cells using nanoliter droplets. *Cell*, **161**(5), 1202–1214.

McGinnis, C. S., Murrow, L. M., and Gartner, Z. J. (2019). DoubletFinder: Doublet detection in single-cell RNA sequencing data using artificial nearest neighbors. *Cell Systems*, **8**(4), 329–337.e4.

Rand, W. M. (1971). Objective criteria for the evaluation of clustering methods. *Journal of the American Statistical association*, **66**(336), 846–850.

Rousseeuw, P. J. (1987). Silhouettes: A graphical aid to the interpretation and validation of cluster analysis. *Journal of Computational and Applied Mathematics*, **20**, 53–65.

Tibshirani, R. (1996). Regression shrinkage and selection via the lasso. *J. Royal Stat. Soc. Series B*, **58**(1), 267–288.

Tsamardinos, I., Aliferis, C. F., and Statnikov, A. (2003). Time and sample efficient discovery of markov blankets and direct causal relations. In *Proceedings of the ninth ACM SIGKDD*. ACM Press.

Tung, P.-Y., Blischak, J. D., Hsiao, C. J., Knowles, D. A., Burnett, J. E., Pritchard, J. K., and Gilad, Y. (2017). Batch effects and the effective design of single-cell gene expression studies. *Scientific Reports*, **7**(1).

Vallejos, C. A., Marioni, J. C., and Richardson, S. (2015). BASiCS: Bayesian analysis of single-cell sequencing data. *PLOS Computational Biology*, **11**(6), e1004333.

Wolock, S. L., Lopez, R., and Klein, A. M. (2019). Scrublet: Computational identification of cell doublets in single-cell transcriptomic data. *Cell Systems*, **8**(4), 281–291.e9.

Zappia, L., Phipson, B., and Oshlack, A. (2017). Splatter: simulation of single-cell RNA sequencing data. *Genome Biology*, **18**(1).

Zheng, G. X., Terry, J. M., Belgrader, P., Ryvkin, P., Bent, Z. W., Wilson, R., Ziraldo, S. B., Wheeler, T. D., McDermott, G. P., Zhu, J., *et al.* (2017). Massively parallel digital transcriptional profiling of single cells. *Nature communications*, **8**, 14049.

Zou, H. and Hastie, T. (2005). Regularization and variable selection via the elastic net. *Journal of the royal statistical society: series B (statistical methodology)*, **67**(2), 301–320.
